# Development and Experimental Validation of a Dry Non-Invasive Multi-Channel Mouse Scalp EEG Sensor through Visual Evoked Potential Recordings

**DOI:** 10.3390/s17020326

**Published:** 2017-02-09

**Authors:** Donghyeon Kim, Chanmi Yeon, Kiseon Kim

**Affiliations:** 1 School of Electrical Engineering and Computer Science, Gwangju Institute of Science and Technology (GIST), Gwangju 61005, Korea; dhkim518@gist.ac.kr; 2 Department of Biomedical Science and Engineering, Gwangju Institute of Science and Technology (GIST), Gwangju 61005, Korea; ycmi0331@gist.ac.kr

**Keywords:** EEG, dry-type, non-invasiveness, multi-channel electrodes, laboratory mouse, visual evoked potential

## Abstract

In this paper, we introduce a dry non-invasive multi-channel sensor for measuring brainwaves on the scalps of mice. The research on laboratory animals provide insights to various practical applications involving human beings and other animals such as working animals, pets, and livestock. An experimental framework targeting the laboratory animals has the potential to lead to successful translational research when it closely resembles the environment of real applications. To serve scalp electroencephalography (EEG) research environments for the laboratory mice, the dry non-invasive scalp EEG sensor with sixteen electrodes is proposed to measure brainwaves over the entire brain area without any surgical procedures. We validated the proposed sensor system with visual evoked potential (VEP) experiments elicited by flash stimulations. The VEP responses obtained from experiments are compared with the existing literature, and analyzed in temporal and spatial perspectives. We further interpret the experimental results using *time-frequency distribution* (TFD) and distance measurements. The developed sensor guarantees stable operations for in vivo experiments in a non-invasive manner without surgical procedures, therefore exhibiting a high potential to strengthen longitudinal experimental studies and reliable translational research exploiting non-invasive paradigms.

## 1. Introduction

Electroencephalography (EEG) from rodents, especially genetically manipulated mice, yields primary information to comprehend neurobiological phenomena [1,2,3]. In recent years, remarkable achievements regarding EEG research using mice have been reported including post-natal development of neuronal networks [4,5], brain disease analysis [6,7,8], pharmacological effects for neuroscience [9,10], translational research and neural engineering. Sensors acquiring EEG signals play a significant role in providing information reflecting electrical activations of neurons in the brain.

A head is structurally divided into several layers: scalp, skull, dura, and brain. The sensor acquiring EEG signals can be located in every layer. The signal characteristics are highly dependent on the invasiveness of the sensor placement [11]. Many research groups have proposed various mouse EEG electrodes positioned on diverse places to understand diverse signals according to brain activation. Such advanced methods like electrodes measuring intracranial EEG [2,12,13,14,15], surface field potential [16], epidural EEG [17], and epicranial EEG [3,18,19,20,21,22] have been proposed to be employed in mice. Many researchers conducted invasive EEG-related studies using laboratory animals with beneficial accessibility for surgical procedures regarding normal and disease models. Laboratory animal-related research not only provides insights on the basics of biology, but also exploits the fundamental principles for human beings and other animal-related applications. For successful translational research, Garner in [23] emphasized preparing the same environment for animal research as we would design for human beings. For the non-invasive EEG, however, the experimental studies using the animals are not prevalent. A novel framework for the non-invasive scalp EEG sensor system targeting laboratory mice would provide a reliable and effective tool for translational research that can be applied to the non-invasive paradigms for human beings and other animals.

In this study, we propose a dry non-invasive multi-channel mouse scalp EEG sensor that is placed on the mouse scalp without undergoing any surgical processes. Insights into the development of the proposed EEG sensor were obtained from the seminal works reported in [24,25], mainly targeting human EEGs. In the former work, the configuration of the electrode surface as multiple spikes was shown to improve the electrical conductivity and mechanical fixation on the scalp. The authors of the latter work showed that electrodes with an internal spring facilitates high geometric conformity between the sensor and the scalp surface such that the skin–electrode interface maintains low impedance. Our mouse EEG sensor is devised by combining these two characteristics, i.e., properties exhibited by spiky contacts and internal springs. Only after shaving the hair off the head can the sensor be utilized to measure mouse brainwaves. The subjects wear the sensor only during the experimental period, and continue to live normally as the sensor is removed after the experiment. This property facilitates longitudinal studies through repetitive experiments without interfering with daily behaviours. The proposed methodology does not require the surgical procedure or conductive gel for signal acquisition. These features confer benefits to save costs preparing the experiments, and provide an environment similar to the advanced human being scalp EEG research. The dry-type sensor prevents the merging of brain signals between adjacent channels due to the wet conductive gel on the small head. From the perspective of experimental results, the analysis through a multi-channel electrode assembly would certify the large-scale network aspects of the brain feasibly. Conclusively, we suggest the scalp EEG research framework targeting the laboratory mouse as an efficient paradigm with a high potential to provide insights on the non-invasive EEG paradigms for humans and other animals such as working animals, pets, and livestock. The work presented here is an extension of a preliminary work reported in [26]. In this paper, the practical usability of the proposed sensor is mainly discussed.

The remainder of this paper is organized as follows. Section 2 describes the proposed mouse EEG sensor with emphasis on its components, structure and applications. As an experimental validation step of the proposed sensor, we demonstrate the sensor with visual evoked potential experiments. Section 3 presents the experimental environments, results, and interpretations for reactions elicited by flash stimulus in spatio-temporal aspects. In Section 4, we additionally depict the acquired signals using *time-frequency distribution* (TFD) and distance measures. Finally, conclusive remarks are provided in Section 5.

## 2. Development of the Mouse EEG Sensor

In this section, we describe the components, structure and usage of the proposed mouse EEG sensor in detail. We explain the features and functionality of the fundamental elements configuring the sensor and the structural properties of the sensor. Finally, a description on how the proposed sensor should be used in animal experiments is given.

### 2.1. Components

**Figure 1:**
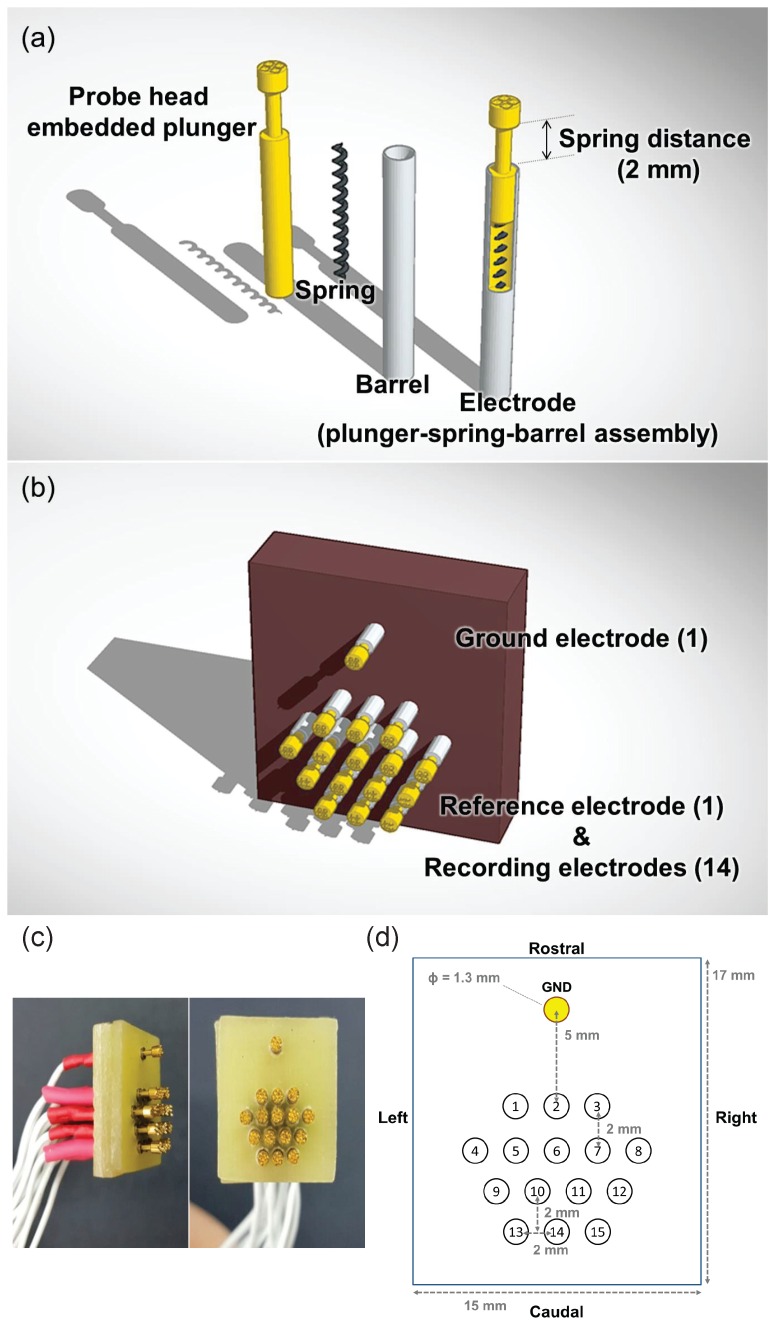
Mouse electroencephalography sensor description: (**a**) three elements composing the electrode, probe head embedded plunger, spring, and barrel (**b**) a schematic design of the proposed mouse EEG sensor (**c**) side and frontal view of the manufactured sensor (**d**) the array of sixteen electrodes; ground electrode (GND) is highlighted in yellow, with fifteen electrodes comprised of one reference electrode and fourteen recording electrodes.

As depicted in Figure 1, the proposed sensor consists of total sixteen electrodes and a substrate panel for fixing the electrodes. Each pin-type electrode is assembled to contain three elements: a probe head embedded plunger, an internal spring, and a barrel as shown in Figure 1a. The probe head embedded plunger only contacts the scalp of the mouse directly to acquire EEG signals. As it is in contact with the skin, high conductivity and bio-compatibility are requisite characteristics. The gold (Au) plated bronze is chosen as the material of the probe to enhance the conductivity for a high quality of EEG signals, and to prevent adverse effects on the skin–electrode interface due to its non-toxic and anti-corrosive features. The diameter of the probe head is 1.3 mm so that multiple pins are positioned on the small animal head. Each tetra-spiked shape of the probe head not only increases sensitivity through a widened contact area, but also distributes the force to reduce pain when the probes are tightly placed on the scalp surface.

The high carbon steel spring which has 20 g of feedback force is located inside each plunger. A sufficiently large spring force is desirable to maintain the appropriate level of electrode–skin contact impedance. The spring force proportionally increases with the spring distance (2 mm). Inside the plunger, the spring has been compressed in advance with a 20% pre-load. The pre-loaded spring enables a certain amount of force to exist prior to the contact between electrode and scalp. The plunger-connected internal spring is completely fixed, pushing back after being located on the scalp.

The barrel holds and supports the plunger. Since it needs to be durable enough for bearing the pressure during the electrode–skin interface, we used a barrel composed of nickel plated beryllium-copper (1% Be–Cu). In addition, the barrel allows the signal acquired by the plunger to travel through it with its high electrical conductivity.

Finally, the pin-type electrode is assembled orderly within the probe head embedded plunger- spring-barrel. Each electrode is linked to the wire with a touch proof connector (Plastics One Inc., Roanoke, VA, USA) to be connected to the amplifier for EEG signal acquisition.

The probe-fixing substrate has a total of sixteen holes to fix sixteen electrodes. The non-conducting plastic substrate serves as an insulator so that the substrate separates the acquired signals from the electrodes. Each electrode functions as a single channel at the designated position. Figure 1b,c illustrate the proposed sensor composed of all the components described so far.

### 2.2. Structure

The array of sixteen electrodes has been designed to measure the EEG signals from different points of the overall brain region. The sixteen electrodes consist of one ground, one reference and fourteen recording electrodes. The ground channel is located on the brow (middle of nose and eyes). All the other electrodes are distributed over the entire head surface at regular intervals. The electrode coordinates are (in mm, anteroposterior/lateral with respect to bregma): +7/0 (ground electrode), +2/−2, +2/0, +2/+2, 0/−4, 0/−2, 0/0 (bregma), 0/+2, 0/+4, −2/−3, −2/−1, −2/+1, −2/+3, −4/−2, −4/0, −4/+2 (reference and recording electrodes) as shown in Figure 1d. All the locations of the electrodes are compared and referred to according to the brain atlas of C57BL/6J [27]. The sensor with multiple channels covers the primary motor cortex in both hemispheres, the primary somatosensory cortex in both hemispheres, cingulate cortex, bregma, parietal association cortex, and visual cortex areas.

One of the fifteen electrodes (channel 1–15), except the ground channel, plays the role of the reference electrode depending on the purpose of the experiment. The electrodes are positioned on the region of interest (ROI) function as recording channels, and the electrode which is located at the furthest location from ROI operates as the reference channel.

Although the multiple electrodes are fixed on the flat insulated plate, internal springs inside the electrodes enable us to cover the whole curvature of the scalp flexibly. Thus, all sixteen electrodes make contact with the scalp closely and the signals from different positions are acquired properly.

### 2.3. Instructions

In order to adopt the proposed sensor for the actual in vivo experiment, maintaining continuous contact between sensor and scalp is essential. To minimize dynamic noise created by movements, we have considered a scenario in which the animal is fixed on the stereotaxic frame.

Though the conductive gel improves signal quality by reducing impedance, we considered a dry-type EEG sensor to avoid potential skin irritations, and to prevent the merging brain signals between adjacent channels due to the wet gel on the small head.

**Figure 2:**
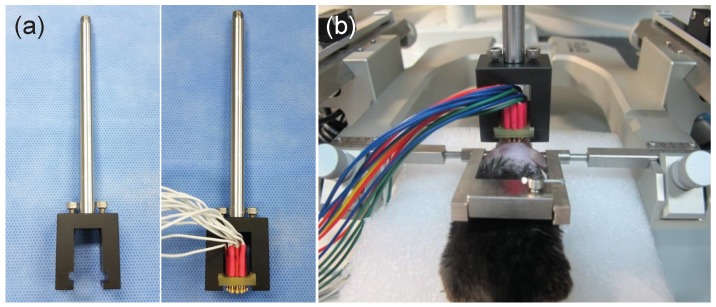
Instructions for using the mouse electroencephalography (EEG) sensor: (**a**) customized sensor holder (**b**) in vivo mouse EEG experiment using the proposed sensor and customized holder on the stereotaxic frame.

As shown in Figure 2a, the customized holder of the sensor is designed to fix the sensor on the scalp of the mouse placed on the stereotaxic frame. The custom-made holder is made of non-conducting plastic to prevent electrical leakage or noise during EEG acquisition. The customized holder hung from the stereotaxic frame enables the sensor to keep the designated position fixed, and thus allows the signal acquisition process to progress steadily. Figure 2b illustrates the sensor placed on the scalp of mouse fixed using the stereotaxic device.

## 3. In Vivo Test: Visual Evoked Potential Experiment

To demonstrate the success of the proposed sensor in measuring mice brainwaves, we conducted the event-related potentials (ERPs) experiment. The ERPs, neural activities triggered by given stimulation, indicate a spatio-temporal brain activity and provide an indicator to confirm changes of brain signals between the pre-stimulation period and the post-stimulation period. As many ERP studies with respect to the mice have been conducted [28,29], we analyse and express the temporal reaction under the stimulation and spatially activated region of brain in this section.

In order to verify the viability of the proposed sensor, a proper stimulation is required to acquire the high quality of brain signals with minimum noise. The physically direct stimulus could incur unexpected responses such as muscle activities contaminating brain waves. In this experiment, we use a flash stimulator that provides indirect visual stimulation. The visual evoked potential (VEP) prevents confusion between the measured brain signals and the adverse noise effects.

Neurophysiological investigation on mouse VEP responses have been widely reported in the literature. The VEP responses originate in the visual cortex, and dark-adapted VEP responses have higher amplitudes and shorter latencies as compared to light-adapted VEP responses [30]. Conventionally, flash and patterned stimulation methods have been prevalently used to elicit VEP responses. Basic VEP properties under the patterned visual stimuli are reported spatially and temporally for studies on plasticity and degeneration/regeneration in the visual system [31]. The effects of dark-rearing are also reported to result in increased latencies and decreased temporal resolution [32]. The VEP experimental procedures facilitate the performance evaluation of the manufactured sensor with respect to previous related works. We focus on the proper operation of the developed sensor by analysing the recorded flash VEP responses reported in the literature.

In this section, we describe the experimental procedures and results of VEP recording elicited by flash stimulator. We explain the experimental set-up and highlight the underlying temporal and spatial features.

### 3.1. Experimental Environments and Apparatus

Eleven male C57BL/6 mice (eight weeks of age, body weight (BW): 22–23 g) were used as subjects for VEP experiments. Mice were anaesthetized with an intra-peritoneal (i.p.) injection of ketamine:xylazine 100:10 (100 mg/mL:10 mg/mL) mixture (10 μL/BW g) during experimental procedures. Hair around the head was removed using a hair-clipper and commercial depilatory (Veet cream, Reckitt Benckiser, Slough, UK) The mouse head and sensor were fixed in the stereotaxic frame. We located the mouse EEG sensor using the custom-made sensor holder and manipulator of the stereotaxic instrument. All the experimental procedures for animal care and handling followed institutional guidelines of Gwangju Institute of Science and Technology (GIST), Gwangju, Korea.

After placing the EEG sensor on the scalp, we put the mouse in a shielded darkroom (width × length × height: 61 × 61 × 60 cm). Prior to the experiment, the mouse was placed in darkroom around 10 min in advance so as to adapt to the darkness. We measured impedance to check the adherence between electrodes and scalp using g.USBamp (g.tec, Schiedlberg, Austria) and BCI2000 software (Schalk lab, Albany, NY, USA) [33]. The impedance values of all the channels were under 1 M-ohm for all mice. Mitsar-EEG 202-24 (MITSAR, Sankt-Peterburg, Russia) amplifier, EEGStudio EEG acquisition software (MITSAR, Sankt-Peterburg, Russia), and Photo Stimulator (MITSAR, Sankt-Peterburg, Russia) were used for the VEP recording. The Photo Stimulator was fixed at a distance of 20 cm in front of the mouse’s eyes. We provided white color flash stimulation, for which illumination with distance 200 mm is 550 ± 20% lx and a flash duration is 10 ms. We provided a flash stimulus and allowed 10 s recovering time for sufficient relaxation during one trial. The VEP experiment for each mouse was repeated for 200 trials. We used channel 2 (Figure 1d) as a reference electrode and measured signals from the remaining fourteen recording channels focusing on the occipital lobe related to the visual cortex area in the caudal direction. The amplifier was operated at a sampling rate of 500 Hz. The collected data were processed with a band pass filter from 5 to 100 Hz and a notch filter (60 Hz).

### 3.2. Experimental Results

In this section, we describe analytical methodology adopted and the experimental results. We compared the acquired VEP responses with known results in previous research. We showed that the developed multi-channel sensor successfully acquires VEP responses by observing a distinct difference in signal shapes between pre-stimulation period and post-stimulation period in the time domain, and by discovering spatially activated brain regions related to the visual cortex area.

#### 3.2.1. Comparison with Existing Results in Literature

A direct comparison between the experimental results and previous works in literature is hindered by several issues. Basically, the research regarding animal scalp VEP responses is rarely reported. In addition, the experimental results vary due to various factors such as experimental protocols, the location of electrodes, anaesthesia, experimental environments, and stimulation parameters including flash rate, intensity of illumination, and duration of the flash.

The current experimental paradigm and protocol is highly similar to [34], which emphasises analysis of the epicranial VEP responses of mice. As in [34], we conducted the VEP experiments using anaesthetized C57BL/6J. The location of the reference electrode is the same as our experiment. In addition, the position for one (channel 14 in Figure 1d) of the multiple electrodes of our developed sensor is identical to the single recording electrode placed on the occipital region in [34]. We acquired non-invasive scalp VEP responses, while [34] inserted an invasive needle electrode beneath the scalp to contact the skull for epicranial VEP recording. The stimulation parameters are slightly different between two experiments. Unlike the photic-stimulator used in our experiment that provides flash stimuli for a duration of 10 ms, [34] used flash stimuli of 10 μs duration. While [34] provided one flash (duration of 10 μs) per one second, we provided one flash (duration of 10 ms) in a 10-second long trial. Hence, Ref. [34] is the most analogous research selected for comparison with our experimental results to verify consistency with the existing literature.

**Figure 3:**
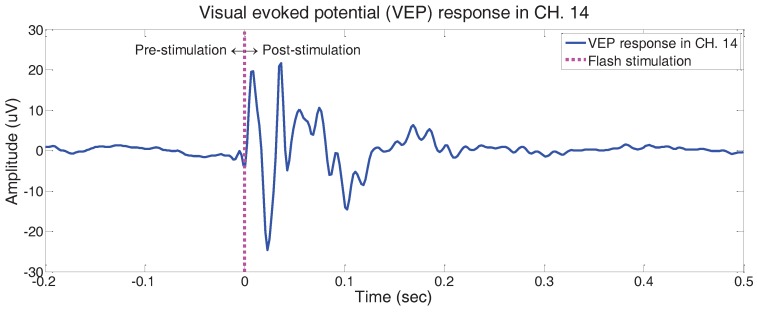
Visual evoked potential experimental results from the occipital area (channel 14): grand averaged visual evoked potential signals of all subjects and all trials from pre-stimulation period (−200 ms) to post-stimulation period (500 ms).

We calculated the ensemble average of VEP responses from eleven mice as shown in Figure 3 and compared it with [34], which computed the grand average VEP signal from fifteen mice. Figure 3 illustrates the VEP responses obtained through our experiment from the pre-stimulation period (−200 ms) to the post-stimulation period (500 ms) as the stimulation is given at time 0. Note that the signal fluctuates only for a while after the stimulation is given, whereas the signal gradually stabilises with time in the post-stimulation period as the pre-stimulation period. Overall patterns and morphologies of the recorded signal reveal similar behaviours as stated in [34], such that the positive and negative alternating peaks are apparent. Although the peak latencies of our dataset appear earlier than that of [34], the peak latency is related with stimulation parameters and experimental environments. Lopez et al. [35] conducted VEP experiments considering unanaesthetized unrestrained mice without scotopic adaptation, and the earlier latencies of peaks observed are compared to [34]. Moreover, [35] emphasized the effects of anaesthesia on electro-physiological responses due to body temperature and cortical depression. Our experimental environments maintained a lower level of anaesthesia compared to [34] with the dark-adaptation process and long-lasting experiments. Hence, the early appearance of peak latencies in our dataset follows the results reported in [35].

Although several variables such as flash intensity, flash rate, and anaesthesia level during the experiments would affect the morphology of the VEP responses, it is noteworthy to exemplify the analogous existing literature for better implications as supportive methods. In general, the high flash intensity affects the high VEP amplitudes [35] and the fast latencies. In addition, the high flash rates make the VEP responses appear late [34]. Moreover, the high anaesthesia level affects the VEP response, incurring a decrease in body temperatures in part [35,36,37]. Our experimental environments involving high flash intensity in dark room, low flash rate, and low anaesthesia levels reveal the early emergence of latencies in the VEP responses. Further research on neurophysiological interpretations would, however, be of great significance.

#### 3.2.2. Topographical Analysis Based on the Peak Latencies

In this section, we describe spatial topographical representations based on the acquired VEP responses from all the multiple recorded channels. The brain structure is divided into several parts, and each manages particular functions including cognition, vision, hearing, motor skills, and language. The performance of the proposed sensor can be evaluated by matching the activated brain regions elicited by an optical stimulus with visual cortex and cognition related areas. A topographical representation of waveforms visualizes the spatial configuration of the surface potentials. The two-dimensional topographical map reflects neuronal activities, providing large-scale network aspects [3]. Specifically, we used the VEP signals during each peak latency interval to observe the brain activation spatially.

Fundamental VEP signal processing procedures were required to treat the raw data for analysis. We first composed two hundred epochs for each mouse from the entire recorded dataset. On the basis of the time points indicating each flash stimulus, the recording from 200 ms before stimulation (pre-stimulation period) to 500 ms after stimulation (post-stimulation period) was extracted as one epoch. We then made the dataset be reference-free using a typical common average reference (CAR) method in the re-reference step. Finally, we computed the grand average over all the trials of all subjects for each electrode. We calculated the grand average VEP responses during the post-stimulation period based on the pre-stimulus baseline period. We further calculated the peak latency intervals. For a total of eleven mice subjects, we analysed the single channel VEP response recorded from the occipital area (channel 14) and assessed latencies of the negative peaks ($N_{1}$, $N_{2}$, and $N_{3}$) and positive peaks ($P_{1}$, $P_{2}$, and $P_{3}$). The subscript index represents the order of the negative/positive peak appearances. We extracted the peaks utilising the band pass filter including theta, alpha, and beta bands, which enhances the isolation of the peaks [38], and then analysed the mean and standard error (SE) to calculate a 95% confidence interval [39].

**Figure 4:**
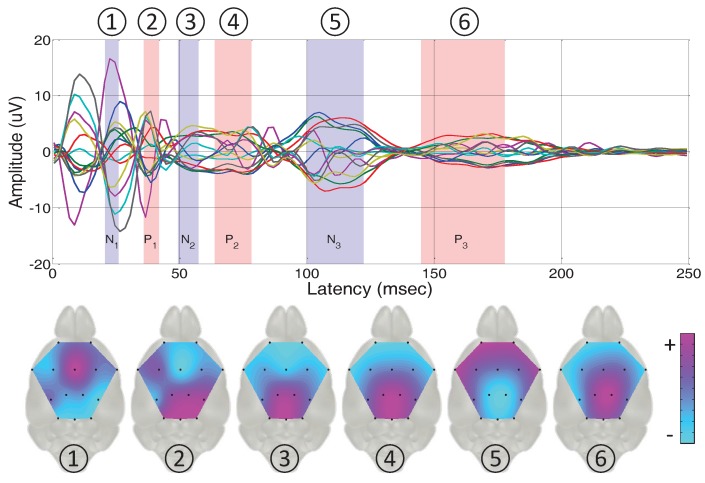
Superimposed visual evoked potential responses from multiple channels with six shaded areas implying a 95% confidence interval of the peak latencies from the channel 14 located on the occipital area (visual cortex area), and six momentary topographical maps representing averaged signals corresponding to each peak latency interval.

Figure 4 illustrates grand average VEP responses of fourteen channels in the time domain. In particular, the 95% confidence intervals of the peak latencies are displayed in the shaded area. For each alternative negative and positive peak interval and each channel, we calculated the mean value to reflect a representative single value for the grand averaged waveforms. We expressed the six momentary maps through EEGLAB software (SCCN, La Jolla, CA, USA) [40] as shown in Figure 4. The topographical images express activations of frontal, central, and occipital regions with high and low variation of fluctuations. The frontal area of the brain manages cognition related function, and the occipital area of the brain is known as the visual cortex area. Through spatial topographical analysis based on the peak latency on the occipital region, we clearly observe brain activation related to the visual cortex area. Therefore, it indicates that we successfully conducted VEP experiments and obtained validated results.

In general, the six maps alternately display positive and negative activations of the visual cortex area in the occipital region compared to other brain regions, and these trends almost follow the peaks from channel 14. The third map corresponding to the second negative peak ($N_{2}$) from channel 14, however, shows positive activation of the occipital area as compared to other brain regions. This indicates that, although the VEP response in a single channel implies a negative peak, its signal value can be higher than signals from other channels. Therefore, the developed sensor has the potential to analyse not only conventional single channel recording, but also multi-channel signals spatially spanning the entire brain.

#### 3.2.3. Multi-Channel VEP Responses in Spatio-Temporal Aspects

A conventional observation of VEP signals in the time domain provides information on whether the subject activated neural responses. Since the time point of stimulus is fixed, we confirm that the developed sensor operates as expected by comparing the brain waveforms in the pre-stimulation and post-stimulation periods.

**Figure 5:**
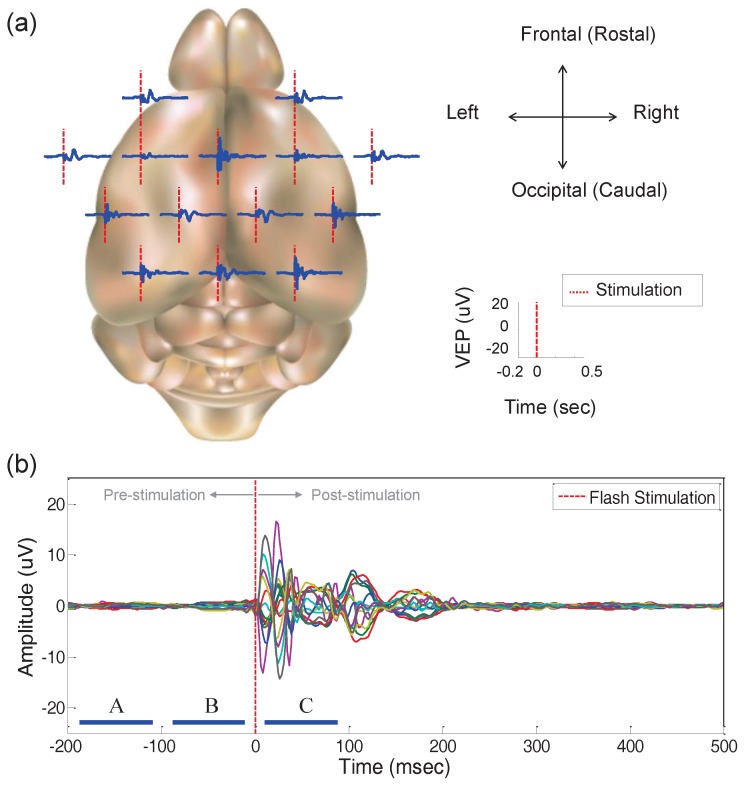
Visual evoked potential (VEP) experimental results: (**a**) grand averaged VEP signals of all subjects and all trials superimposed on the mouse brain surface; (**b**) superimposed VEP responses in the time domain, and three time segments indicated as *A* (−200∼−100 ms), *B* (−100∼0 ms), and *C* (0∼100 ms).

We display the grand averaged waveforms of all the channels superimposed on a mouse brain surface template as shown in Figure 5a. The VEP responses measured from the entire head surface region appear to exhibit several characteristics. Firstly, the brain waves of the left hemisphere and right hemisphere are symmetric. Furthermore, the brain signals in the frontal, central, and occipital area represent similar patterns in the time domain. It can be deduced that several brain regions in the coronal direction are associated with the VEP responses, not partially activated in the left or right hemisphere. Figure 5b illustrates the superimposed signals of all the channels. Brain waveforms after flash stimulation revealed distinct reactions compared to the brain signals in the pre-stimulation period. As the fluctuations gradually stabilized, the subject progressively relaxed with time. We thus clearly observe the difference between pre-stimulation and post-stimulation periods in the temporal domain.

## 4. Analysis of VEP Responses in the Time-Frequency Domain

Signal analysis including both temporal and spectral aspects provides insights to comprehend various facets of the signal. Specifically, a distance measure provides a quantitative indicator expressing differences between signals numerically. Such distance values can be applied to effectively represent signal changes in two different time segments as numerical quantities, and can be further used for statistical data processing.

We verified the experimental validation of the developed sensor through a conventionally typical analysis of VEP responses in temporal and spatial aspects previously in Section 3. In this section, we describe the acquired signals in the time-frequency domain, and we apply a distance measure to interpret the results in different perspectives and to compare with the preceding section. First, we interpret the acquired VEP responses regarding temporal and spectral aspects simultaneously by applying *time-frequency distribution* (TFD). We also calculated the difference of brainwaves between pre-stimulation period and post-stimulation period using the distance measure adapted to the TFD. We visually displayed the changes of brainwaves in two states using a topographical map in terms of the distance values.

### 4.1. Time-Frequency Distribution (TFD) Analysis

A variety of signals could be expressed as TFD, which is defined as an energy density function of both time and frequency [41]. Born–Jordan distribution (BJD), which has an attractive property of reduced interference distribution (RID) [42], was used to represent our dataset as TFD.

**Figure 6:**
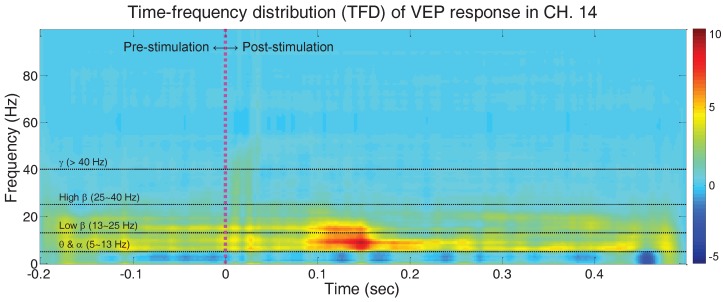
VEP response from single channel (channel 14): grand average time-frequency distribution (TFD) of VEP regarding all subjects and all trials. Born–Jordan distribution is used as TFD.

In Section 3.2.1, the grand average of VEP responses from eleven mice is calculated and discussed. While Figure 3 illustrates the VEP responses in the conventional temporal aspect, Figure 6 depicts the VEP responses from channel 14 that are positioned on the occipital area in temporal and spectral aspects simultaneously. We computed BJD for each trial of the experiments for all of the mice, followed by the grand average calculation of the BJD from the eleven subjects. Figure 3 and Figure 6 represent the same signal from two different perspectives: time domain and time-frequency domain. As an impulse-shaped signal appears immediately after the flash stimulation, we observe the activation in the entire frequency range momentarily after stimulation. We also confirm that the theta, alpha and low beta bands around 0.1∼0.2 s after stimulation reveal the highest activation. This moment is related with $N_{3}$ and $P_{3}$ latency as described earlier in Section 3.2.2, and Figure 4.

### 4.2. Topographical Analysis Using TFD-Based Distance

The distance measure adapted to TFD provides a proper approach to characterize the event related potentials [43]. The advantage of the distance measure for TFD is that it not only quantifies the difference between signals as a single number, but also considers both temporal and spectral features simultaneously [43]. We calculated the Kolmogorov distance adapted to TFD [43] so as to quantify the difference of signals expressed in BJD. Kolmogorov distance is one of the popular distance measures functioning to compute the difference between two distributions, and Ref. [43] successfully analysed and interpreted ERP signals using Kolmogorov distance adapted to TFD.

We split the waveforms into three intervals of 100 ms. Based on time point zero at which the stimulation was given, the durations were separated and denoted by segment *A* (−200∼−100 ms), *B* (−100∼0 ms), and *C* (0∼100 ms) as shown in Figure 5b. The time segments *A* and *B* are the pre-stimulus periods, while the segment *C* is the post-stimulus period. For each mouse, the BJDs of the VEP dataset were computed for each trial and each channel. All the BJDs were averaged over two hundred trials indicating brainwaves in time segments *A*, *B*, and *C* with respect to each mouse and each channel. We denote the averaged BJD of each mouse and each channel as $C_{S}^{m,ch}\left(t,\omega \right)$ (*m*: mouse index from one to eleven, ch: recording channel index from one to fourteen, *S*: time segments *A*, *B*, or *C*). With respect to these BJDs, we calculated the Kolmogorov distance to quantify the differences between two BJDs as the single number as shown below:

(1) $$d_{S_{1}S_{2}}^{m,ch}=\;\int \int \;\left|NC_{S_{1}}^{m,ch}\left(t,\omega \right)-NC_{S_{2}}^{m,ch}\left(t,\omega \right)\right|dtd\omega ,$$

where

(2) $$NC_{S}^{m,ch}\left(t,\omega \right)=\frac{\left|C_{S}^{m,ch}\left(t,\omega \right)\right|}{\;\int \int \;\left|C_{S}^{m,ch}\left(u,v\right)\right|dudv}.$$

The Kolmogorov distances are calculated based on the normalized TFD, $NC_{S}^{m,ch}\left(t,\omega \right)$. The distance reveals the changes in pairwise time segments. In our dataset, the pairwise time segments $S_{1}S_{2}$ are AB, AC and BC, i.e., there are three kinds of distance values, $d_{AB}^{m,ch}$, $d_{AC}^{m,ch}$, and $d_{BC}^{m,ch}$, corresponding to 14 electrode channels (ch = 1–14) with 11 mice (*m* = 1–11).

**Figure 7:**
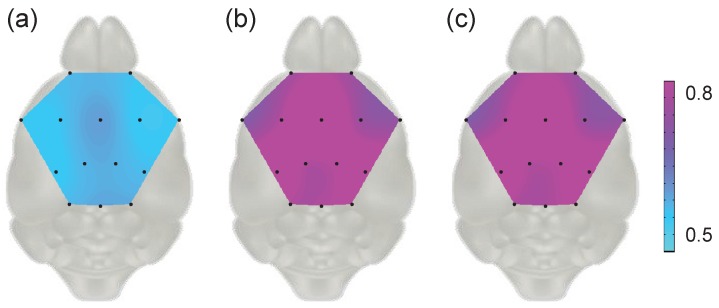
Grand average of Kolmogorov distances adapted to *time-frequency distributions* (Born–Jordan distribution) with respect to eleven mice: (**a**) Kolmogorov distance between $C_{A}^{m,ch}\left(t,\omega \right)$ and $C_{B}^{m,ch}\left(t,\omega \right)$; (**b**) Kolmogorov distance between $C_{A}^{m,ch}\left(t,\omega \right)$ and $C_{C}^{m,ch}\left(t,\omega \right)$; (**c**) Kolmogorov distance between $C_{B}^{m,ch}\left(t,\omega \right)$ and $C_{C}^{m,ch}\left(t,\omega \right)$.

We displayed grand averaged distance values of all the subjects for each channel in topographic maps as shown in Figure 7. We found the variations of brainwaves in the pre-stimulus period were consistent among all the channels showing low distance values (Figure 7a), while the change of signals with stimulation revealed high distance values (Figure 7b,c). Figure 7 illustrates the temporal and spatial traits of the experimental results. The obvious difference between high distance values implying the change after stimulation and low distance values implying change without any stimulation reveals the temporal feature similar to that in Section 3.2.3. In addition, the distinct pattern dividing the brain region such as the occipital visual cortex related area and the frontal cognitive related area indicates brain activation regarding flash stimulation, which is in agreement with Section 3.2.2.

## 5. Conclusions

We presented the process of design, fabrication and experimental validation of a novel mouse scalp EEG sensor possessing traits of multi-channel, dry-type, and non-invasiveness. The multi-channel electrodes provide high spatio-temporal resolution to comprehend the brain activities in large-scale networks. The dry-type feature enables the sensor to acquire the EEG signals without conductive gel, which saves the cost of preparing experiments and prevents merging the brain signals between adjacent channels. The non-invasive scalp sensor for laboratory mice provides opportunities to conduct research directly applicable to human and other animal non-invasive scalp EEG related studies.

We verified the practical validation through visual evoked potential (VEP) experiments, showing the consistency of our results with those in the previous study. Changes in brainwaves after a stimulation compared to those in the pre-stimulation period were confirmed in the time domain. Additionally, we spatially observed the activation of the occipital area related with the visual cortex through topographical map analysis. Furthermore, we interpreted recorded VEP responses by applying time-frequency distribution (TFD) and distance measures in different aspects, in order to quantify the brainwave changes. By describing the VEP responses in the time-frequency domain, we found the highly activated components in temporal and spectral aspects simultaneously.

Straightforward and simple usability of the developed sensor would increase the accessibility to animal brain research. Although we provided instructions for the developed sensor with respect to the anaesthetized animal, adopting the non-invasive sensors for freely moving animals can be another issue for further investigation. However, the proposed sensor and instruction may also have potential in various research topics including EEG-based anaesthesia monitoring systems and assessments of reactions under the different parameters of the stimulation. It is noteworthy that the research scope of our proposed sensor would further span the longitudinal studies with respect to the laboratory animals as well as the reliable translational research for human being and other animal (including laboratory animals, working animals, pets, and livestock) scalp EEG related fields exploiting the non-invasive paradigms.

## Abbreviations

The following abbreviations are used in this manuscript:

BJD	Born–Jordan distribution
CAR	Common average reference
EEG	Electroencephalography
ERP	Event related potential
RID	Reduced interference distribution
ROI	Region of interest
SE	Standard error
TFD	Time-frequency distribution
VEP	Visual evoked potentia
